# Choice of futility boundaries for group sequential designs with two endpoints

**DOI:** 10.1186/s12874-017-0387-4

**Published:** 2017-08-08

**Authors:** Svenja Schüler, Meinhard Kieser, Geraldine Rauch

**Affiliations:** 1 Institute of Medical Biometry and Informatics, University of Heidelberg, Im Neuenheimer Feld 130.3, Heidelberg, 69120 Germany; 2 Institute of Medical Biometry and Epidemiology, University Medical Center Hamburg Eppendorf, Martinistr. 52, Hamburg, 20246 Germany

**Keywords:** Group sequential design, Stopping for futility, Two endpoints, Intersection-union test

## Abstract

**Background:**

In clinical trials, the opportunity for an early stop during an interim analysis (either for efficacy or for futility) may relevantly save time and financial resources. This is especially important, if the planning assumptions required for power calculation are based on a low level of evidence. For example, when including two primary endpoints in the confirmatory analysis, the power of the trial depends on the effects of both endpoints and on their correlation. Assessing the feasibility of such a trial is therefore difficult, as the number of parameter assumptions to be correctly specified is large. For this reason, so-called ‘group sequential designs’ are of particular importance in this setting. Whereas the choice of adequate boundaries to stop a trial early for efficacy has been broadly discussed in the literature, the choice of optimal futility boundaries has not been investigated so far, although this may have serious consequences with respect to performance characteristics.

**Methods:**

In this work, we propose a general method to construct ‘optimal’ futility boundaries according to predefined criteria. Further, we present three different group sequential designs for two endpoints applying these futility boundaries. Our methods are illustrated by a real clinical trial example and by Monte-Carlo simulations.

**Results:**

By construction, the provided method of choosing futility boundaries maximizes the probability to correctly stop in case of small or opposite effects while limiting the power loss and the probability of stopping the study ‘wrongly’. Our results clearly demonstrate the benefit of using such ‘optimal’ futility boundaries, especially compared to futility boundaries commonly applied in practice.

**Conclusions:**

As the properties of futility boundaries are often not considered in practice and unfavorably chosen futility boundaries may imply bad properties of the study design, we recommend assessing the performance of these boundaries according to the criteria proposed in here.

**Electronic supplementary material:**

The online version of this article (doi:10.1186/s12874-017-0387-4) contains supplementary material, which is available to authorized users.

## Background

In recent years, the flexibility and efficiency of clinical trials became increasingly important, in particular for trials from the pharmaceutical industry. Group sequential designs give the opportunity to stop the study early during an interim analysis, thereby saving time and financial resources. Generally, the study can either be stopped for efficacy if the study goal is prematurely achieved or for futility, if reaching the aim of the trial seems desperate. Decision rules to stop a trial early for efficacy have been broadly investigated. Since the pioneering works of Pocock [1] and O’Brien and Fleming [2], these designs have been implemented since long in clinical trial routine. A comprehensive overview and general concepts of the statistical methodology in group sequential designs are provided by Jennison and Turnbull [3]. With respect to futility stopping, there are mainly two fundamental approaches in the statistical literature [3, 4]. The first approach is a conditional one, where the study is stopped for futility, if the conditional power falls under a prespecified threshold. This conditional approach can further be divided into stochastic curtailment [5, 6], a frequentist approach, and methods based on the predictive power or the predictive probability [4], which are partially or fully Bayesian methods [7, 8]. The second approach is to define futility stopping boundaries either in isolation or in conjunction with group sequential efficacy boundaries [3]. The choice of adequate boundaries to stop the study early for futility has been discussed only briefly in these works. This is astonishing as unfavorable futility boundaries may have serious consequences with respect to the performance of the study design.

In our work, we propose a general method for the construction of futility boundaries. In addition, we introduce several intuitive criteria which should be considered when defining ‘optimal’ futility boundaries described as follows. First, unless offset by increased sample size, early stopping for futility reduces the overall power. The aim is thus to avoid a too high loss in power. Second, the study should be stopped early for futility if the unknown true effect is far away from the anticipated effect under the alternative hypothesis (‘correctly’ stopping for futility). Finally, if the unknown true effect corresponds to the anticipated effect under the alternative hypothesis, the study should not be stopped for futility (‘wrongly’ stopping for futility).

In principle, these optimality criteria might be applied in any two-stage design. In the literature, group sequential designs with futility stopping are usually formulated for a single primary endpoint [9–12]. Beside several works on interim efficacy evaluation in group sequential designs with multiple endpoints [13–18] there are only few works suggesting group sequential methods for multiple endpoints including futility stopping [19, 20]. However, we believe that such designs are of particular relevance for clinical trials with multiple primary endpoints for the reason specified in the following. When considering two endpoints, the power of the trial depends on the expected effects of both endpoints and on the correlation between them. Assessing the feasibility of the trial is therefore a particular challenge, as the number of required parameter values to be correctly specified in the planning stage is large. Therefore, the option for an early futility stop is attractive to account for these uncertainties. Moreover, the required sample size for a clinical trial with several primary endpoints is usually higher than for a single primary endpoint and therefore the need to stop the trial early in case of unfavorable outcomes is of particular importance.

In this work, we apply the general construction method of futility boundaries to three different two-stage group sequential designs for two endpoints. For these designs, we propose an algorithm to choose ‘optimal’ futility boundaries with respect to the above mentioned criteria. Thereby as Jennison and Turnbull [3], we define group sequential futility stopping boundaries in conjunction with efficacy boundaries.

This paper is organized as follows. The “Methods” section introduces first the study design, the test problem and general principles of group sequential designs and then describes the new approach of choosing ‘optimal’ futility stopping boundaries as motivated above. Moreover, different group sequential designs for two endpoints profiting from these optimal futility boundaries are proposed. Furthermore, the derivation of ‘optimal’ futility boundaries is described. The “Results” section investigates the performance characteristics of the new methods by means of Monte-Carlo simulations cased on a real clinical trial example. Finally, we conclude with a discussion.

## Methods

Standard group sequential methods allow stopping a study early at an interim analysis where the outcome is observed and analyzed for a part of the maximum number of patients. The study can thereby either be stopped for efficacy or for futility. For simplicity, we restrict our considerations to a controlled clinical trial with one interim analysis and one final analysis resulting in a group sequential design with two stages. In this section, we first consider the situation of a single endpoint in order to describe the new approach of choosing futility boundaries generally. After that, group sequential designs for two endpoints are proposed.

### Test problem and general principles

The (local) test problem for the endpoint under investigation is given by

1 $$ H_{0}^{\delta} : \theta \leq \delta \qquad \text{ versus} \qquad H_{1}^{\delta} : \theta > \delta, \qquad \delta \leq 0,  $$

where *δ*=0 implies a one-sided superiority test problem and *δ*<0 corresponds to a test for non-inferiority. Considering binary or continuous outcome measures, *θ* is given by the absolute risk difference or mean difference, respectively, whereas for time-to-event data *θ* indicates the logarithm of the hazard ratio. The standard approaches to test for superiority are given by the chi-square test for binary data, the *t*-test for continuous data and the log-rank test for time-to-event data [21]. Note that all these test statistics are approximately standard normally distributed under the corresponding null hypothesis.

In a two-stage group sequential design the test statistics *T*_1_ and *T*_2_ used at the interim analysis and at the final analysis, respectively, corresponds to the standard test statistics of a fixed design using all data collected so far with some modifications for the case of time-to-event data [22]. The correlation between the normally distributed test statistics *T*_1_ and *T*_2_ then exclusively depends on the (assumed) information fraction at interim which can be specified in the planning stage. After the correlation between the test statistics has been determined, the adjusted (one-sided) local significance levels *α*_1_ and *α*_2_ can be defined by taking this correlation into account. The local significance levels in a group sequential design can be chosen in various ways, e.g. as constant or increasing in time [1, 2, 23–25]. Then the null hypothesis given in (1) is rejected at interim whenever the one-sided *p*-value *p*_1_ referring to *T*_1_ fulfills *p*_1_≤*α*_1_. The null hypothesis is rejected at the final analysis whenever the one-sided *p*-value *p*_2_ corresponding to *T*_2_ fulfills *p*_2_≤*α*_2_.

In general, stopping for futility without compromising the type I error is possible at any time and independent of any predefined rules as an early acceptance of *H*_0_ decreases the actual type I error rate. In the context of group sequential designs, it can generally be differentiated between binding and non-binding stopping for futility rules, compare also Bretz *et al* [26]. ‘Binding’ means that stopping for futility at the interim analysis is obligatory whenever the futility criteria are met. When the data suggest stopping for futility, it is thus *not* allowed to continue the trial for other external reasons. If a binding futility rule is applied, the local significance levels can be increased in order to fully exhaust the global significance level which is otherwise no longer guaranteed as futility stopping implies a lower probability of rejecting the null hypothesis. In contrast, the non-binding version does not commit early futility stopping. Therefore, there may be situations were the data advise stopping for futility, but the study is continued nonetheless for other reasons, e.g. as new external information suggests that the futility criteria might be too strong. As a consequence, the local significance levels cannot be adjusted and the global significance level is not fully exhausted. In clinical trial applications non-binding futility boundaries are usually applied because they allow reacting flexibly to interim results such as adverse events or new external information. However, quantifying the performance properties (in terms of power loss or ‘correctly’ and ‘wrongly’ stopping for futility) of non-binding rules is impossible as the study progress is not predictable from the observed effect at interim.

For the reasons specified above, we will focus in this work on binding stopping criteria at interim but without increasing the local significance levels. In general, a futility rule can equivalently be expressed either in terms of a boundary for the test statistic or as an upper bound for the *p*-value. We will use the latter approach without loss of generality, that is the study is stopped for futility at interim whenever *p*_1_>*α*_*f*_, where *α*_*f*_ is the futility boundary.

### Defining optimal binding futility boundaries

Choosing adequate futility boundaries is an important challenge as unfavorable futility boundaries may have serious consequences with respect to the performance of the study design. In case of ‘strong’ futility boundaries, for example, if the study is stopped for futility whenever the one-sided *p*-value is larger than 0.2, the overall power loss can be large and the study might be stopped for futility in too many situations caused by only small but non-relevant deviations from the planning assumptions. In such cases, the probability of ‘wrongly’ stopping for futility is high. In the case of ‘liberal’ futility boundaries, given, for example, as a lower bound for the *p*-value of 0.8, the overall power loss is quite small but at the same time small or opposite effects often do not result in an early stop for futility. Hence, the probability of ‘correctly’ stopping for futility is low in this case [9]. The idea of ‘optimal’ futility boundaries proposed here is to provide a high rate of ‘correctly’ stopping for futility and to simultaneously restrict the loss in power and the rate of ‘wrongly’ stopping for futility. To provide ‘optimal’ futility boundaries in this sense, an ‘admissibility condition’ is defined as follows.

#### **Definition 1**

(***βγ***-admissible futility boundaries) Let $H_{0}^{\delta }:\theta \leq \delta $ denote the one-sided null hypothesis of the corresponding test problem and $H_{1,\theta _{1}}^{\delta }$ be the alternative hypothesis for a given effect *θ*_1_>*δ*, for which the trial should have power 1−*β*∈[0,1] given an overall significance level of *α*. Let *β*_*l*_∈[0,1] be the acceptable overall power loss in a group sequential design with a binding stopping for futility rule and let *γ*∈[0,1] denote the acceptable probability of stopping for futility under $H_{1,\theta _{1}}^{\delta }$, the so called ‘wrongly’ stopping for futility rate. Then a futility boundary *α*_*f*_ is called ***βγ*** **-admissible** if the following conditions are met:

- 1. $P_{H_{1,\theta _{1}}^{\delta }}(H_{0}^{\delta }\,$ is rejected in stage 1) + $P_{H_{1,\theta _{1}}^{\delta }}(H_{0}^{\delta }$ is rejected in stage 2 and $H_{0}^{\delta }$ is neither rejected nor accepted in stage 1) ≥1−*β*−*β*_*l*_,
- 2. $P_{H_{1,\theta _{1}}^{\delta }}$(The study is stopped for futility based on *α*_*f*_ in stage 1) ≤*γ*,

where $P_{H_{1,\theta _{1}}^{\delta }}(\cdot)$denotes the probability under the assumption that $H_{1,\theta _{1}}^{\delta }$ holds true.

For predefined values of *β*_*l*_ and *γ* there generally exist several *β* *γ*-admissible futility boundaries. These boundaries differ in the probability of ‘correctly’ stopping for futility as there is no condition on exhausting the admissible power loss *β*_*l*_ or the probability of ‘wrongly’ stopping for futility *γ*.

In order to determine ‘optimal’ futility boundaries, the probability of early stopping for futility should be preferably high in case of a small or opposite effect which deviates considerably from the anticipated treatment effect *θ*_1_. This motivates the following definition.

#### **Definition 2**

(***βγ***-optimal futility boundaries) Let $H_{0}^{\delta }:\theta \leq \delta $ denote the one-sided null hypothesis of the corresponding test problem and $H_{1,\theta _{1}}^{\delta }$ be the alternative hypothesis for a given effect *θ*_1_>*δ*, for which the trial should have power 1−*β*∈[0,1] given an overall significance level of *α*. Let $A_{\beta _{l},\gamma }$ denote the set of all *β* *γ*-admissible futility boundaries for a maximally admissible power loss *β*_*l*_∈ [ 0,1] and a maximally admissible ‘wrongly’ stopping for futility rate *γ*∈ [ 0,1]. Let *θ* ^∗^<*θ*_1_ denote the largest effect under $H_{1}^{\delta }$ for which stopping the study for futility would still be considered as ‘correct’ and let $H_{1,\theta ^{*}}^{\delta }$ denote the corresponding alternative hypothesis. Then the futility boundary $\phantom {\dot {i}\!}\alpha _{f}\in A_{\beta _{l},\gamma }$ which maximizes the probability of stopping for futility under $H_{1,\theta ^{*}}^{\delta }$ given as

$$\begin{aligned} \alpha_{opt}&=\max_{\left\{\alpha_{f}\in A_{\beta_{l},\gamma}\right\}}\\&P_{H_{1,\theta^{*}}^{\delta}}(\text{The study is stopped for futility based on}\; \alpha_{f} \text{in stage} 1) \end{aligned} $$

is called ***βγ*** **-optimal**, where $P_{H_{1,\theta ^{*}}^{\delta }}(\cdot)$ denotes the probability under the assumption that $H_{1,\theta ^{*}}^{\delta }$ holds true.

The *β* *γ*-optimal futility boundary defines a lower bound for the *β* *γ*-admissible boundaries, as all boundaries that are larger than the *β* *γ*-optimal boundary automatically meet the *β* *γ*-admissible conditions. Note that for group sequential designs for a single endpoint (as well as for designs with two endpoints we consider in this work) the optimal futility boundary could also be determined by maximizing the probability of correctly stopping under the null hypothesis effect *θ* instead of under *θ* ^∗^. However, in more complex multiple endpoint group sequential designs this monotonicity property might no longer hold true. The derivation of *β* *γ*-optimal futility boundaries will be described below in “Derivation of the local significance levels and *βγ*-optimal futility boundaries” section.

In general, the optimality of group sequential designs is usually assessed by means of the average sample size, where a low average sample size is preferable. Although the average sample size is a common criteria to judge the performance of a group sequential design, it has also major shortcomings when applied as the unique measure of performance. For example, the power loss of a group sequential design compared to a correctly specified single-stage design is a further performance criterion. Liu et al. proposed a performance score combining both criteria (average sample size and power loss) [27]. The application of the performance score for the situation of an intersection-union test is, e.g., provided by Kieser et al. [28]. Despite these important new aspects discussed in literature, there further remain some open topics with respect to a performance assessment of a group sequential design: The average sample size is a summary measure which does not necessarily show the true sample sizes because the variability of the sample size is completely ignored. Therefore, a low average sample size is only a good optimality criteria if the variability of the sample size is also low. Instead of looking at the average sample size and its variability, an alternative approach could be to judge the correctness of early stopping (implying a low sample size) or continuing (implying a high sample size). The latter is what we have investigated in our work.

### Investigated group sequential designs with futility stop based on two endpoints

Motivated by the fact that the application of adequate binding futility boundaries is of particular interest when analyzing several endpoints, we consider three different group sequential designs that incorporate two endpoints and different futility stopping rules. The aim is to define a test procedure which offers a maximal gain in information from two endpoints of interest but simultaneously requires a minimal number of patients to save resources, especially when the effects are lower than originally anticipated. These specific two-stage designs will subsequently be used to illustrate the impact of *β* *γ*-optimal futility boundaries. In the following, the indexes *EP*_1_ and *EP*_2_ will denote the affiliation to the two endpoints under investigation.

For Approaches 1 and 2, the aim is to show a significant effect in both endpoints which are then commonly referred to co-primary endpoints. In this case, the test hypotheses can be formulated using the intersection-union test principle

2 $$\begin{array}{*{20}l} \textstyle H_{0}^{IUT, \delta_{1}, \delta_{2}} &: H_{0}^{EP_{1},\delta_{1}} \cup H_{0}^{EP_{2}, \delta_{2}}\ \text{versus} \end{array} $$

3 $$\begin{array}{*{20}l} H_{1}^{IUT, \delta_{1}, \delta_{2}} &: H_{1}^{EP_{1},\delta_{1}} \cap H_{1}^{EP_{2}, \delta_{2}},\qquad\quad \delta_{1}, \delta_{2} \leq 0, \end{array} $$

where the local test hypotheses are given as stated in (1). The most rigorous requirement for a clinical trial with two primary endpoints is to base the efficacy proof on demonstrating superiority for both equivalently relevant endpoints ($H_{0}^{IUT, 0, 0}$ versus $H_{1}^{IUT, 0, 0}$). In the case that the endpoints are of different relevance, e.g. an efficacy and a safety endpoint, a less rigorous test procedure may also be appropriate. Therefore, Approach 2 combines a superiority test for the efficacy endpoint with a non-inferiority test for the safety endpoint or the endpoint of less clinical relevance ($H_{0}^{IUT, 0, \delta _{2}}$ versus $H_{1}^{IUT, 0, \delta _{2}},\ \delta _{2}<0$). Note that for the ease of representation, it is assumed without loss of generality that the efficacy endpoint corresponds to *EP*_1_. A group sequential test procedure including binding futility stopping rules for $H_{0}^{IUT,0,0}$ or $H_{0}^{IUT,0,\delta _{2}}$, respectively, is defined as follows.

**Approach 1 (** $H_{0}^{IUT,0,0}$ **) and Approach 2 (** $H_{0}^{IUT,0,\delta _{2}},\delta _{2}<0$ **)**

- Stage 1: ∙ The study is stopped early with rejection of $H_{0}^{IUT,\delta _{1},\delta _{2}}$ if $p_{1}^{EP_{1}} \leq \alpha _{1}^{EP_{1}}$ and $p_{1}^{EP_{2}} \leq \alpha _{1}^{EP_{2}}.$
- ∙ The study is stopped early for futility (with acceptance of $H_{0}^{IUT,\delta _{1},\delta _{2}}$) if $p_{1}^{EP_{1}} \ge \alpha _{f}^{EP_{1}}$ or $p_{1}^{EP_{2}} \ge \alpha _{f}^{EP_{2}}.$
- Stage 2: At the final analysis, $H_{0}^{IUT,\delta _{1},\delta _{2}}$ is rejected if $p_{2}^{EP_{1}} \leq \alpha _{2}^{EP_{1}}$ and $p_{2}^{EP_{2}} \leq \alpha _{2}^{EP_{2}}.$ Otherwise $H_{0}^{IUT,\delta _{1},\delta _{2}}$ is accepted.

When an efficacy and a safety endpoint are considered it is not necessarily required to perform a hypothesis test for the safety endpoint. But even in this case, the option to stop for futility can be based on both endpoints so that small or opposite effects in the safety endpoint can additionally be ruled out. Approach 3 therefore considers a situation were one endpoint is formally tested for superiority at interim and at the final analysis and the other is solely used as an additional criterion for futility stopping. Thus, while the efficacy assessment is exclusively based on endpoint 1, stopping for futility at interim can be based on endpoint 1 *or* on endpoint 2. A further situation to apply Approach 3 could be when a short-term surrogate is used to assess the futility of the trial and a long-term efficacy endpoint is used to assess the efficacy at the final analysis. For example, in oncology trials a common surrogate for overall survival is given by progression-free survival which provides more events in a shorter observational time-frame. The test problem is then given by

4 $$ H_{0}^{EP_{1},0} : \theta \leq 0 \qquad \text{ versus} \qquad H_{1}^{EP_{1},0} : \theta > 0,  $$

which corresponds to the definition given in (1) with *δ*=0 and the related group sequential procedure is defined as follows.

**Approach 3 (** $H_{0}^{EP_{1},0}$ **)**

- Stage 1: ∙ The study is stopped early with rejection of $H_{0}^{EP_{1},0}$ if $p_{1}^{EP_{1}} \leq \alpha _{1}^{EP_{1}}.$
- ∙ The study is stopped early for futility if $p_{1}^{EP_{1}} \ge \alpha _{f}^{EP_{1}}$ or $p_{1}^{EP_{2}} \ge \alpha _{f}^{EP_{2}}.$
- Stage 2: At the final analysis, $H_{0}^{EP_{1},0}$ is rejected if $p_{2}^{EP_{1}} \leq \alpha _{2}^{EP_{1}}.$ Otherwise $H_{0}^{EP_{1},0}$ is accepted.

Unlike for the Approaches 1 and 2, the efficacy proof is now only based on *EP*_1_. However, the other endpoint *EP*_2_ still influences the study result, as early stopping for futility due to *EP*_2_ is possible.

### Derivation of the local significance levels and *βγ*-optimal futility boundaries

As motivated above, we do not increase the local significance levels to fully exhaust the type I error. Therefore, the local levels can be chosen as usual in group sequential designs without taking futility stopping into account, e.g. constant or increasing [1,2]. The derivation of increased local significance levels in conjunction with futility stopping (in order to exhaust the overal significance level) is described in the Supplementary Material (see Additional file 1).

To calculate the *β* *γ*-optimal futility boundaries for a specific study situation, we implemented a search algorithm in R which at first determines all constellations of futility boundaries for both endpoints which simultaneously maximally exhaust the prespecified thresholds for the probability of ‘wrongly’ stopping for futility *γ* and for the power loss *β*_*l*_. Note that these are the *β* *γ*-admissible boundaries. Subsequently the constellations of boundaries which maximize the probability of ‘correctly’ stopping for futility are determined, which are the *β* *γ*-optimal boundaries.

## Results

To illustrate application of the proposed methods, the three group sequential designs incorporating two endpoints are applied (by simulations) to a real clinical trial example. For these three designs, *β* *γ*-admissible and *β* *γ*-optimal futility boundaries are calculated and furthermore investigated in terms of overall power and probability of ‘correctly’ and ‘wrongly’ stopping for futility.

### Clinical trial example and simulation design

The RENAAL study was a randomized, double-blind, placebo-controlled trial conducted to investigate the effect of losartan on renal and cardiovascular outcomes in patients with type 2 diabetes and nephropathy [29]. The primary outcome was a time-to-first event composite endpoint, where the events correspond to doubling the baseline serum creatinin concentration, end-stage renal disease, and death. The recruitment time was fixed to 2 years and the minimal follow-up duration to 3.5 years. Patients were allocated to the placebo and the intervention group in a 1:1 ratio. The original planning assumptions used for sample size calculation were given by estimated 5 year event rates of 0.58 and 0.464 in the placebo and the intervention group, respectively [30]. Assuming constant hazard functions over time (exponentially distributed survival times), the underlying hazards can be directly calculated from the given event rates [31]. The resulting hazard ratio for the composite endpoint is given by $\lambda _{CE}^{C}/ \lambda _{CE}^{I}=0.0145/0.0104=1.394$, wherethe parameter *λ* denotes the hazard function, which is assumed to be constant here, the index *CE* stands for ‘composite endpoint’ and the group affiliation is expressed by the indexes *I* and *C* for the intervention and the control group, respectively.

The clinically most relevant component of the composite endpoint defined above is clearly given by death. Within the context of a composite endpoint, current guidelines on clinical trials systematically recommend to investigate the components of a composite endpoint separately, in particular the most relevant components [32–34]. Therefore, it might provide a relevant gain in information to include this endpoint in the analysis strategy instead of exclusively considering the composite endpoint. The two endpoints under consideration are thus given by a composite endpoint combining doubling of baseline serum creatinin concentration, end-stage renal disease, and death and by the endpoint death alone. Thus, whenever a death occurs, this corresponds to an event in both endpoints. However, the composite endpoint consist of more events of other types. This illustrates that the two endpoints are correlated by construction. The assumed event rates for the endpoint death have not been published. For the sake of illustration, we assume a hazard ratio for death given by $\lambda _{MC}^{C}/\lambda _{MC}^{I}=0.01/0.0074=1.351$, where the index *MC* stands for ‘main component’.

The presented example is appealing in the sense that the two endpoints correspond to a composite endpoint and a main component. In the specific case of a composite endpoint, any additional confirmatory information on the components provides an important gain in information. Therefore, application of all three group sequential approaches presented in this manuscript can be illustrated by means of a unique example. In most other clinical trial applications only one of the proposed test problems fits the specific confirmatory requirements.

#### ‘Correctly’ and ‘wrongly’ stopping for futility

In order to differentiate between ‘correctly’ and ‘wrongly’ stopping for futility, we modify the underlying hazard assumptions. Consequently, we consider different hazard ratios for the main component that deviate from the original planning assumption given by $\lambda _{MC}^{C}/ \lambda _{MC}^{I}=0.01/0.0074=1.351$. Of course, deviations from the planning assumptions could also occur in the composite endpoint or in both endpoints. As every event in the main component also corresponds to an event in the composite endpoint and for sake of an easier illustration, we restrict our considerations to deviations in the main component. The following 7 hazard ratio scenarios for the main component are considered {1.351;1.3;1.25;1.2;1.15;1.1;1.05}, where without loss of generality the hazard in the intervention group was fixed to $\lambda _{MC}^{I}=0.0074$ and deviations in the hazard ratio are due to variations from the assumptions for $\lambda _{MC}^{C}$. Scenario 1 corresponds to the original planning assumptions for the main component while the remaining Scenarios 2 to 7 correspond to decreasing treatment effects in the main component. To determine *β* *γ*-optimal futility boundaries according to Definition 2, the largest effect under the alternative hypothesis has to be determined for which stopping the study for futility would still be considered as ‘correct’. In the RENAAL study, a hazard ratio of $\lambda _{MC}^{C}/\lambda _{MC}^{I}=1.2$ might be a reasonable choice for the main component. Consequently, mis-specifications of the hazard ratio given in Scenarios 2 and 3 might be acceptable, whereas for Scenario 4 to 7 it would be justified to stop the study early for futility. This threshold should be based on aspects of clinical relevance and should be discussed with clinical experts. Note that we base for sake of simplicity the threshold for ‘correctly’ stopping for futility in this example only on deviations from the planning assumptions in the main component. This threshold can also depend on the composite endpoint or even on both endpoints.

#### Sample size considerations

On the way to determine *β* *γ*-optimal futility boundaries and especially to assess the overall power loss *β*_*l*_, at first the reference group sequential design without stopping for futility has to be fixed. For this illustrating example, we apply a two-stage group sequential design where the interim analysis is performed after an anticipated information fraction of 0.5 and where the local significance levels are adjusted according to Pocock [1]. Moreover, we assume that patient recruitment is stopped during the interim analysis. All these settings could be chosen differently and are only of illustrative purpose here. We assume a target power of *β*=0.90 and an overall one-sided significance level of *α*=0.025 for the reference design without stopping for futility. The required sample sizes for these reference designs are then given by 1260 (630 per group) for Approach 1 and by 820 (420 per group) for the less stringent Approach 2 where the main component is tested for non-inferiority with a non-inferiority margin of 0.9 in terms of the hazard ratio. The correlation of the test statistics are then given by *r*_1_=0.83 and *r*_2_=0.82 for Approach 1 and Approach 2, respectively. For Approach 3, testing exclusively the composite endpoint yields a sample size of 730 (365 per group). An analytical derivation of the required sample sizes and the correlations between the test statistics is difficult and the results are thus based on simulations. More details on the sample size derivations are described in the Supplementary Material (see Additional file 2).

### Simulation results

For each scenario described above, we simulated 10.000 data sets and applied the corresponding group-sequential designs. From these, rates of overall power and probability of wrongly or correctly stopping for futility, respectively, are estimated for different constellations of stopping for futility boundaries. The power loss is given as the anticipated power minus the observed overall power. The rates of early stopping in Scenario 1 (according to the planning assumptions) or Scenario 4 lead to the probability of wrongly or correctly stopping for futility, respectively. For varying constellations of futility boundaries the corresponding values of overall power and probability of stopping for futility differ consequentially. A search algorithm (compare “Defining optimal binding futility boundaries” section) finally chooses the constellation of futility boundaries yielding an acceptable power loss while minimizing wrongly stopping for futility. This algorithm can be obtained from the authors on request.

#### Optimal to non-optimal futility boundaries

In order to calculate the *β* *γ*-admissible and *β* *γ*-optimal futility boundaries the maximal acceptable power loss with stopping for futility is set to *β*_*l*_=0.05 and the admissible ‘wrongly’ stopping for futility rate is chosen as *γ*=0.025.

For Approach 1, futility bounds given by $\alpha _{f}^{CE}=0.43$ and $\alpha _{f}^{MC}=0.44$ maximize the ‘correctly’ stopping for futility rate at 0.117. In Approach 2, the *β* *γ*-optimal futility boundaries are given by $\alpha _{f}^{CE}=0.65$ and $\alpha _{f}^{MC}=0.37$ with a probability of ‘correctly’ stopping for futility of 0.087. Approach 3 yields *β* *γ*-optimal boundaries of $\alpha _{f}^{CE}=0.69$ and $\alpha _{f}^{MC}=0.65$ with a probability of ‘correctly’ stopping for futility of 0.085.

#### Comparison of design performance between optimal and non-optimal futility boundaries

As a reference, we compare the *β* *γ*-optimal futility bounds with a common futility boundary of $\alpha _{f}^{CE}=\alpha _{f}^{MC}=0.5$ which is often applied in practice. For Approach 1 and Approach 2 this choice is more liberal than the *β* *γ*-optimal boundaries. Therefore, the probability of ‘correctly’ stopping for futility is smaller with 0.09<0.117 for Approach 1 and 0.047<0.087 for Approach 2. Neither the probability of ‘wrongly’ stopping for futility (given as 0.016 for Approach 1 and 0.018 for Approach 2) nor the overall power loss (given as 0.017 for Approach 1 and 0.006 for Approach 2) exhaust the maximal admissible values *γ*=0.025 and *β*_*l*_=0.05. However, a common futility bound of 0.5 is contained in the set of admissible boundaries for the first two approaches. For Approach 3 the reference futility boundaries correspond to stricter values than the *β* *γ*-optimal ones. Therefore, the probability of ‘correctly’ stopping for futility is higher with 0.158>0.085 but to the prize of a higher probability of ‘wrongly’ stopping for futility with 0.06 which exceeds the admissible value *γ*=0.025 considerably. At the other hand the overall power loss of 0.04 does not exhaust the maximal admissible value *β*_*l*_=0.05.

#### Impact of the planning assumptions on power loss and probability of stopping for futility

**Fig. 1:**
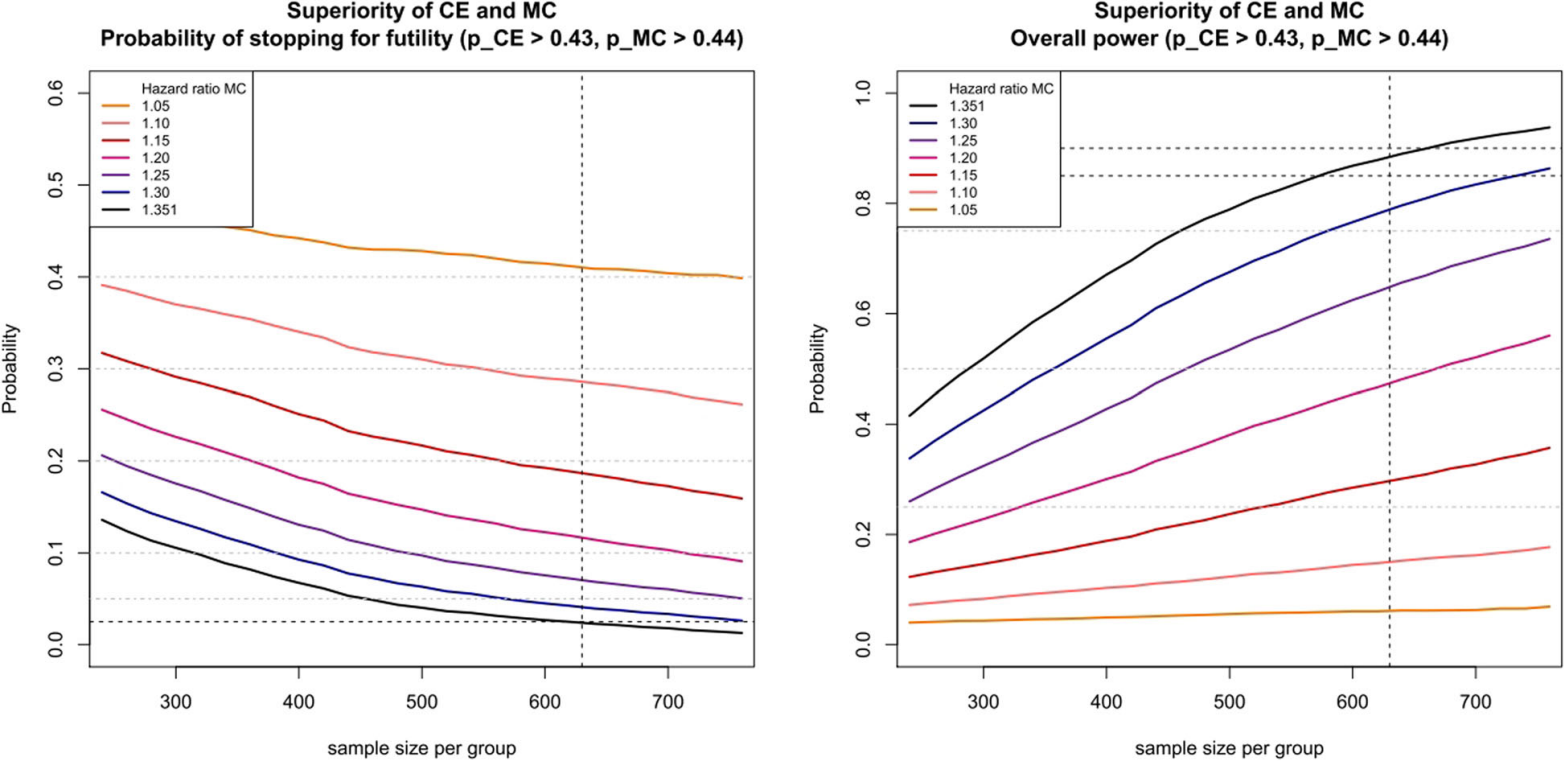
Probability of stopping for futility (*left figure*) and overall power (*right figure*) for Approach 1 using the futility *β* *γ*-boundaries $\alpha _{f}^{CE}=0.43$ and $\alpha _{f}^{MC}=0.44$ for composite endpoint and main component, respectively. The *vertical dotted line* shows the sample size required to achieve a power of 0.9 for the reference design without futility stopping. *Horizontal dotted lines* indicate the thresholds for the rate of ’wrongly’ stopping for futility (*left figure*) and for the admissible power loss (*right figure*), respectively

For Approach 1, Fig. 1 illustrates the dependency of the probability of stopping for futility (left plot) and the corresponding overall power (right plot) as function of sample size per group for the different hazard ratio scenarios of the main component given above for the *β* *γ*-optimal futility boundaries defined above. The differently colored lines in Fig. 1 match the seven hazard ratio scenarios, where the black line corresponds to the original planning assumption.

The right plot shows the overall power where for the *β* *γ*-optimal futility boundaries the line of the original assumption (black line) should not fall below the admissible power 1−*β*−*β*_*l*_=0.90−0.05=0.85 (horizontal dotted line) for a sample size of 630. Decreasing the sample size or decreasing the hazard ratio (which corresponds to a larger deviation from the original planning assumptions) results in a monotone loss in overall power. Note that the global power depends on the correlation of the two test statistics. If the correlation is unknown in the planning stage, we recommend investigating the power by simulation. In general, the power increases with increasing absolute value of the correlation.

The left plot of Fig. 1 displays the probability of stopping for futility. In case of the original planning assumption (black line), the probability of ‘wrongly’ stopping for futility is given by 0.025 for *n*_*group*_=630. As a hazard ratio of 1.2 was considered as the largest effect under the alternative hypothesis for which stopping the study for futility would still be considered as correct, the probability of ‘correctly’ stopping for futility is given by 0.117. Decreasing the sample size or decreasing the hazard ratio (which corresponds to a larger deviation from the original planning assumptions) results in a monotone increase of the probability of stopping for futility. Note that for decreasing probability of ‘wrongly’ stopping for futility the probability of ‘correctly’ stopping for futility also decreases.

For Approach 2, the corresponding plots look similar, but additionally the non-inferiority margin of the main component mainly influences the required sample size. For Approach 3, the displayed curves are much closer to each other, which means that on the one hand the loss in power is less prominent but on the other hand the probabilities of ‘correctly’ stopping for futility are also smaller. Figures for Approach 2 and Approach 3 are provided as Supplementary Material (see Additional files 3 and 4).

#### Influence of the choice of futility boundaries on power and probability of stopping - Admissible and optimal futility boundaries

**Fig. 2:**
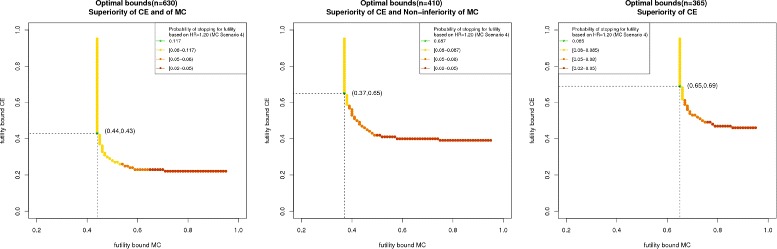
Lower bounds for *β* *γ*-admissible futility boundaries (*yellow*, *orange* and *red dots*) and *β* *γ*-optimal futility boundaries (*green dot* with coordinates as indicated by the *dotted lines*) for the composite endpoint and the main component for Approach 1 (*left plot*), Approach 2 (*middle plot*), and Approach 3 (*right plot*)

Figure 2 shows the constellations of futility boundaries which meet the admissible condition (Definition 1) for Approach 1 (left plot), Approach 2 (middle plot), and Approach 3 (right plot). As for larger futility boundaries the overall power increases and the probability of ‘wrongly’ stopping for futility decreases, all boundary constellations right hand from the curves also fulfill the admissible condition. However, the probability of ‘correctly’ stopping for futility also decreases with increasing futility boundaries. The *β* *γ*-optimal pair of futility boundaries for the composite endpoint and the main component must be an element of the plotted curve, but not all elements of the curve are *β* *γ*-optimal. Colors from yellow to green show increasing probabilities of ‘correctly’ stopping for futility. In all three approaches, the left- hand side of the curves show higher probabilities of ‘correctly’ stopping for futility, whereas for increasing futility boundaries in the main component this probability decreases.

Approach 3 shows the largest futility boundaries as the efficacy proof is based only on the composite endpoint and the overall power loss is thus most vulnerable to decreasing futility boundaries. Comparing the *β* *γ*-optimal futility boundaries of the first two approaches, Approach 2 shows a larger value for the composite endpoint but a smaller for the main component. This is due to the fact that basing the efficacy proof on a non-inferiority test of the main component but on superiority for the composite assigns a higher impact to the composite endpoint. Consequently, the overall power loss is more vulnerable to a decreasing futility boundary in the composite endpoint. For the specific clinical trial situation at hand, all approaches show medium and similar ‘correctly’ stopping for futility rates but Approach 1 by far requires the largest sample size. Approach 2 clearly provides more gain in information compared to Approach 3 as both endpoints are included in the efficacy claim. For this reason, Approach 2 should be recommended here.

## Discussion

By construction, the provided method of choosing futility boundaries maximizes the probability of detecting small or opposite effects while limiting the power loss and the probability of stopping the study ‘wrongly’. The simulation results provided in “Simulation results” section clearly demonstrate the benefit of using such ‘optimal’ futility boundaries in the considered scenarios, especially compared to a futility bound of *α*_*f*_=0.5 which is commonly applied in practice. However, these criteria are generally applicable to any type of group sequential designs. In order to determine the most suitable two-stage design out of the three approaches presented in “Investigated group sequential designs with futility stop based on two endpoints” section, we recommend to compare the performance properties of the designs under *β* *γ*-optimal futility boundaries, which usually differentiate between the designs. The design is then chosen by considering the specific study situation, the gain in information provided by the design, and the probability of ‘correctly’ stopping for futility under the *β* *γ*-optimal futility boundaries.

In this work we investigated binding futility boundaries in order to quantify the performance properties of the corresponding group sequential designs. However, the resulting performance properties of a binding rule can also be used to approximate the performance properties of more liberal non-binding rules as some deviations from the binding rule will not importantly influence the performance.

The performance investigations and calculations of the *β* *γ*-optimal futility boundaries in “Clinical trial example and simulation design” section were done within the context of a composite endpoint and one relevant main component which refers to a situation of two correlated endpoints. We considered only deviations from the original planning assumptions in the main component, which is motivated by the fact that every event in the main component corresponds to an event in the composite endpoint. Similar investigations could be made for deviations in the other endpoint or simultaneous deviations in both endpoints. Generally, our proposed designs could equivalently be applied to endpoints with other, potentially differing, scale levels. In particular, it would also be of interest to consider the performance properties of our designs for uncorrelated endpoints. A systematic investigation of all possible sequential designs and endpoint settings within the current work was not feasible. Therefore, we encourage to perform further simulations in order to determine a suitable two-stage design for the specific situation at hand. Further, in this work we chose the Pocock approach for the local significance levels, which facilitates stopping the study early at the interim analysis. Allowing decision criteria that are more conservative in stopping at the interim analysis as, for example, proposed by O’Brien and Fleming [2] will improve the overall power.

The general principles of optimal futility boundaries might be transferred similarly to group-sequential designs with more than two stages or patient-wise interim looks as long as the number of interim looks is fixed in advance. The implementation of our ideas in these situations will be the task of future work.

## Conclusion

In this paper, we presented general optimality criteria for the choice of suitable futility boundaries which maximize the probability of detecting small or opposite treatment effect while limiting the power loss and the probability of stopping the study ‘wrongly’. We illustrated the criteria on three different group sequential designs including two endpoints, which are motivated by the fact that in many clinical trial applications it is not sufficient to consider only one primary endpoint in order to adequately describe the efficacy of a new treatment. As the properties of futility boundaries are often not considered in practice and unfavorably chosen futility boundaries may have serious consequences with respect to the performance of the study design, we recommend assessing the impact of these boundaries according to the proposed admissibility and optimality criteria.

## Additional files

Additional file 1 Derivation of local significance levels in case of binding futility boundaries. Details on the derivation of local significance levels in case of binding futility boundaries. (PDF 201 kb)

Additional file 2 Sample size calculation of the reference design. Details on the sample size calculation of the reference design for the clinical trial example. (PDF 66 kb)

Additional file 3Probability of stopping for futility and overall power for Approach 2. Plot of probability of stopping for futility (left figure) and overall power (right figure) for Approach 2 using the futility *β* *γ*-boundaries $ \alpha_{f}^{CE}=0.65$ and $ \alpha_{f}^{MC}=0.37$ for composite endpoint and main component, respectively. (JPG 238 kb)

Additional file 4Probability of stopping for futility and overall power for Approach 3. Plot of probability of stopping for futility (left figure) and overall power (right figure) for Approach 3 using the futility *β* *γ*-boundaries $ \alpha_{f}^{CE}=0.69$ and $ \alpha_{f}^{MC}=0.65$ for composite endpoint and main component, respectively. (JPG 202 kb)
