# Supplementary material for: Choice of futility boundaries for group sequential designs with two endpoints
Source: BMC Med Res Methodol. 2017 Aug 8;17:119. doi: 10.1186/s12874-017-0387-4 (PMC5549398; doi:10.1186/s12874-017-0387-4)
Supplement: Supplementary file 1 — Derivation of local significance levels in case of binding futility boundaries. Details on the derivation of local significance levels in case of binding futility boundaries. (PDF 201 kb) [file 12874_2017_387_MOESM1_ESM.pdf]

1 Additional File 1
Derivation of local significance levels in case of binding futility boundaries

As indicated in Section 2.1, using binding stopping for futility boundaries in group sequential designs allows to test each individual null hypothesis with an enlarged local significance level. For Approaches 1 and 2, the following equation has to be solved to determine these local significance levels $\alpha_k^{EP_i},\ i,k=1,2$,

$$\begin{aligned}
&P_{H_0^{IUT,\delta_1,\delta_2}}\Big(H_0^{IUT,\delta_1,\delta_2} \text{ is rejected in stage } 1\Big) + \\
&P_{H_0^{IUT,\delta_1,\delta_2}}\Big(H_0^{IUT,\delta_1,\delta_2} \text{ is rejected in stage } 2 \\
&\qquad\qquad \textit{and } H_0^{IUT,\delta_1,\delta_2} \text{ is neither rejected nor accepted in stage } 1\Big) = \alpha \\
&\Leftrightarrow \\
&P_{H_0^{IUT,\delta_1,\delta_2}}\Big(p_1^{EP_1} \le \alpha_1^{EP_1} \wedge p_1^{EP_2} \le \alpha_1^{EP_2}\Big) + \\
&P_{H_0^{IUT,\delta_1,\delta_2}}\Big(\Big(p_2^{EP_1} \le \alpha_2^{EP_1} \wedge p_2^{EP_2} \le \alpha_2^{EP_2}\Big) \\
&\qquad \wedge\Big(p_1^{EP_1} < \alpha_f^{EP_1} \wedge p_1^{EP_2} < \alpha_f^{EP_2} \wedge \Big(p_1^{EP_1} > \alpha_1^{EP_1} \vee p_1^{EP_2} > \alpha_1^{EP_2}\Big)\Big)\Big) = \alpha,
\end{aligned} \tag{1}$$

where for Approach 1 the global null hypothesis is given as $H_0^{IUT,0,0}$ and for Approach 2 by $H_0^{IUT,0,\delta_2},\ \delta_2 < 0$. To solve equation (1), the correlation between the test statistics of the two endpoints must be known which is unrealistic in practice. A conservative approach for the derivation of adequate local levels is to make use of the property that for the type I error control in the intersection-union test, it is sufficient to control the type I error for each individual hypothesis [1]. This allows separate calculation of the local significance levels for each endpoint by solving the equation

$$\begin{aligned}
&P_{H_0^{EP_i,\delta_i}}\Big(H_0^{EP_i,\delta_i} \text{ is rejected in stage } 1\Big) + \\
&P_{H_0^{EP_i,\delta_i}}\Big(H_0^{EP_i,\delta_i} \text{ is rejected in stage } 2 \\
&\qquad\qquad \textit{and } H_0^{EP_i,\delta_i} \text{ is neither rejected nor accepted in stage } 1\Big) = \alpha \\
&\Leftrightarrow \\
&P_{H_0^{EP_i,\delta_i}}\Big(p_1^{EP_i} \le \alpha_1^{EP_i}\Big) + \\
&P_{H_0^{EP_i,\delta_i}}\Big(p_2^{EP_i} \le \alpha_2^{EP_i} \wedge \alpha_1^{EP_i} < p_1^{EP_i} < \alpha_f^{EP_i}\Big) = \alpha,\ i =, 1, 2,
\end{aligned} \tag{2}$$

were the local test problem is given as

$$H_0^{EP_i,\delta_i} : \theta \le \delta_i \qquad \text{versus} \qquad H_1^{EP_i,\delta_i} : \theta > \delta_i,\ \delta_i \le 0,\ i = 1, 2. \tag{3}$$

Equation (2) can be solved uniquely for $\alpha_k^{EP_i},\ i,k=1,2$, given fixed $\alpha_f^{EP_i},\ i=1,2$, and assuming $\alpha_1^{EP_i} = \alpha_2^{EP_i},\ i=1,2$, where the latter assumption of equal local levels across stages corresponds to the commonly applied approach of Pocock [2]. Note that all equations can equivalently be solved for other group sequential designs,

Table 1 Approaches 1 and 2 - resulting constant local significance levels across stages ($\alpha_1^{EP_i} = \alpha_2^{EP_i}$) for each $EP_i, i = 1, 2$, based on different futility boundaries $\alpha_f^{EP_i}$ for a global one-sided significance level $\alpha = 0.025$ and an assumed information fraction $\pi = 0.5$ at interim

$\alpha_f^{EP_i}$	1	0.9	0.8	0.7	0.6	0.5	0.4	0.3
$\alpha_k^{EP_i}$	0.0147	0.0147	0.0147	0.0147	0.0147	0.0148	0.0149	0.0151

i.e. using the approach by O'Brien and Fleming [3]. However, the local significance levels determined by solving equation (2) will only guarantee that

$$P_{H_0^{IUT,\delta_1,\delta_2}}\left(H_0^{IUT,\delta_1,\delta_2} \text{ is rejected in stage } 1\right) + P_{H_0^{IUT,\delta_1,\delta_2}}\left(H_0^{IUT,\delta_1,\delta_2} \text{ is rejected in stage } 2 \; \textit{and} \; H_0^{IUT,\delta_1,\delta_2} \text{ is neither rejected nor accepted in stage } 1\right) \leq \alpha.$$

In case of no stopping for futility, that is $\alpha_f^{EP_i} = 1, i = 1, 2$, the resulting local levels for an assumed information fraction $\pi = 0.5$ at interim are given by $\alpha_k^{EP_i} = 0.0147,\ i, k = 1, 2$. The local levels are monotonically increasing when lower futility boundaries are chosen. For a fixed futility boundary of $\alpha_f^{EP_2} = 1$ and decreasing values of $\alpha_f^{EP_1}$, the local levels are given by $\alpha_k^{EP_i} = 0.0148,\ i, k = 1, 2$, for $\alpha_f^{EP_1} = 0.5$ and by $\alpha_k^{EP_i} = 0.0151,\ i, k = 1, 2$, for $\alpha_f^{EP_1} = 0.3$.

For Approach 3, the efficacy proof is based on testing EP_1 only with the additional possibility to stop the study for futility based on EP_2. The test hypotheses for EP_1 are thus given as in (3) with $\delta_1 = 0$ ($H_0^{EP_1,0}$ versus $H_1^{EP_1,0}$). To determine the local significance levels $\alpha_k^{EP_1},\ k = 1, 2$, the following equation has to be solved for predefined fixed $\alpha_f^{EP_i},\ i = 1, 2$:

$$P_{H_0^{EP_1,0}}\left(H_0^{EP_1,0} \text{ is rejected in stage } 1\right) + P_{H_0^{EP_1,0}}\left(H_0^{EP_1,0} \text{ is rejected in stage } 2 \; \textit{and} \; H_0^{EP_1} \text{ is neither rejected nor accepted in stage } 1 \; \textit{and} \; H_0^{EP_2,0} \text{ is not accepted in stage } 1\right) = \alpha$$

$$\Leftrightarrow$$

$$P_{H_0^{EP_1,0}}\left(p_1^{EP_1} \leq \alpha_1^{EP_1}\right) + P_{H_0^{EP_1,0}}\left(p_2^{EP_1} \leq \alpha_2^{EP_1} \wedge \alpha_1^{EP_1} < p_1^{EP_1} < \alpha_f^{EP_1} \wedge p_1^{EP_2} < \alpha_f^{EP_2}\right) = \alpha. \quad (4)$$

Table 1 shows the resulting local significance levels for Approaches 1 and 2 for $EP_i, i = 1, 2$, for different futility boundaries $\alpha_f^{EP_i}, i = 1, 2$ (which are obtained by solving equation (2)). Thereby, remember that for Approaches 1 and 2 the local levels for EP_1 and EP_2 are calculated separately [1]. Within this work, we focus on Pocock boundaries [2] and hence the stagewise significance levels are equal $\alpha_1^{EP_i} = \alpha_2^{EP_i}, i = 1, 2$.

Table 2 shows the resulting local significance levels for Approach 3 corresponding to EP_1 for different combinations of futility boundaries for EP_1 and EP_2 (which are obtained by solving equation (4)). The local significance levels for Approach

Table 2 Approach 3 - resulting constant local significance levels across stages $\alpha_1^{EP_1} = \alpha_2^{EP_1}$ for EP_1 based on different futility boundaries $\alpha_f^{EP_1}$, $\alpha_f^{EP_2}$ for a global one-sided significance level $\alpha = 0.025$ and an assumed information fraction $\pi = 0.5$ at interim

	$\alpha_f^{EP_1}$							
$\alpha_f^{EP_2}$	1	0.9	0.8	0.7	0.6	0.5	0.4	0.3
1	0.0147	0.0147	0.0147	0.0147	0.0147	0.0148	0.0149	0.0151
0.9	0.0154	0.0154	0.0154	0.0154	0.0154	0.0154	0.0155	0.0158
0.8	0.0161	0.0161	0.0161	0.0161	0.0161	0.0161	0.0162	0.0165
0.7	0.0168	0.0168	0.0168	0.0169	0.0169	0.0169	0.0170	0.0173
0.6	0.0177	0.0177	0.0177	0.0177	0.0177	0.0178	0.0179	0.0180
0.5	0.0186	0.0186	0.0186	0.0186	0.0186	0.0187	0.0188	0.0190
0.4	0.0197	0.0197	0.0197	0.0197	0.0197	0.0197	0.0198	0.0200
0.3	0.0208	0.0208	0.0208	0.0208	0.0208	0.0208	0.0209	0.0211

3 are generally larger than those for Approaches 1 and 2. This is due to the fact that these local levels are exhaustive solutions of equation (4) as compared to the conservative solutions for Approaches 1 and 2.

Note that the local significance levels for Approaches 1 and 2 (Table 1) equal the first row of the local significance levels of Approaches 3 (Table 2). This is due to the fact that regarding the calculation of the local significance levels, Approaches 1 and 2 are special cases of Approach 3 as for $\alpha_f^{EP_2} = 1$ equation (4) reduces to equation (2) (as a bound of $\alpha_f^{EP_2} = 1$ corresponds to no stopping for futility with the probability of an early acceptance of $H_0^{EP_2,0}$ being equal to 0).

The corresponding `R` code for deriving the local significance levels for the different approaches can be obtained from the authors on request.

Author details

References

1. Berger, R.: Multiparameter hypothesis testing and acceptance sampling. Technometrics **24**, 295–300 (1982)
2. Pocock, S.: Group sequential methods in the design and analysis of clinical trials. Biometrika **64**(2), 191–199 (1977)
3. O'Brien, P., Fleming, T.: A multiple testing procedure for clinical trials. Biometrics, 549–556 (1979)
