# Supplementary material for: Choice of futility boundaries for group sequential designs with two endpoints
Source: BMC Med Res Methodol. 2017 Aug 8;17:119. doi: 10.1186/s12874-017-0387-4 (PMC5549398; doi:10.1186/s12874-017-0387-4)
Supplement: Supplementary file 2 — Sample size calculation of the reference design. Details on the sample size calculation of the reference design for the clinical trial example. (PDF 66 kb) [file 12874_2017_387_MOESM2_ESM.pdf]

1 Additional File 2
Sample size calculation of the reference design

We begin with the simulation of the required sample size for the three approaches by considering the situation that no stopping for futility is implemented ($\alpha_f^{CE} = \alpha_f^{MC} = 1$). The sample size calculations are all based on a group sequential design with two stages, with an interim analysis at an information fraction $\pi = 0.5$, and with an anticipated power of 0.9 to detect the assumed treatment effects using a one-sided overall significance level of $\alpha = 0.025$. Our simulations were performed with the software `R`. To determine the required sample sizes in the corresponding group sequential designs, we use a search algorithm which computes the power values for given maximal sample sizes until a power of 0.9 is reached. Thereby, for each sample size $10,000$ runs are performed. When performing group sequential designs with survival data, it is generally possible either to stop the recruitment at interim until all patients have been observed for the planned minimal follow-up time, or to continue recruitment. The latter approach, which is also implemented in the software `ADDPLAN`, seems more realistic in clinical practice, as a recruitment stop is often not realizable in clinical trial routine. However, a clear drawback is, that the interim time point must be chosen during the recruitment period to provide a possible advantage in terms of preventing an unnecessarily high sample size. An interim analysis during the limited recruitment period, however, can result in a relatively low information fraction which might question a meaningful early decision. For this reason, we decided to stop the recruitment during the interim analysis in our simulation. Therefore, our results are not directly comparable to those of group sequential designs calculated with the software `ADDPLAN`. The general performance characteristics of the proposed methods, however, are not influenced by this simulation strategy.
