# Supplementary figures and images for: Choice of futility boundaries for group sequential designs with two endpoints
Source: BMC Med Res Methodol. 2017 Aug 8;17:119. doi: 10.1186/s12874-017-0387-4 (PMC5549398; doi:10.1186/s12874-017-0387-4)

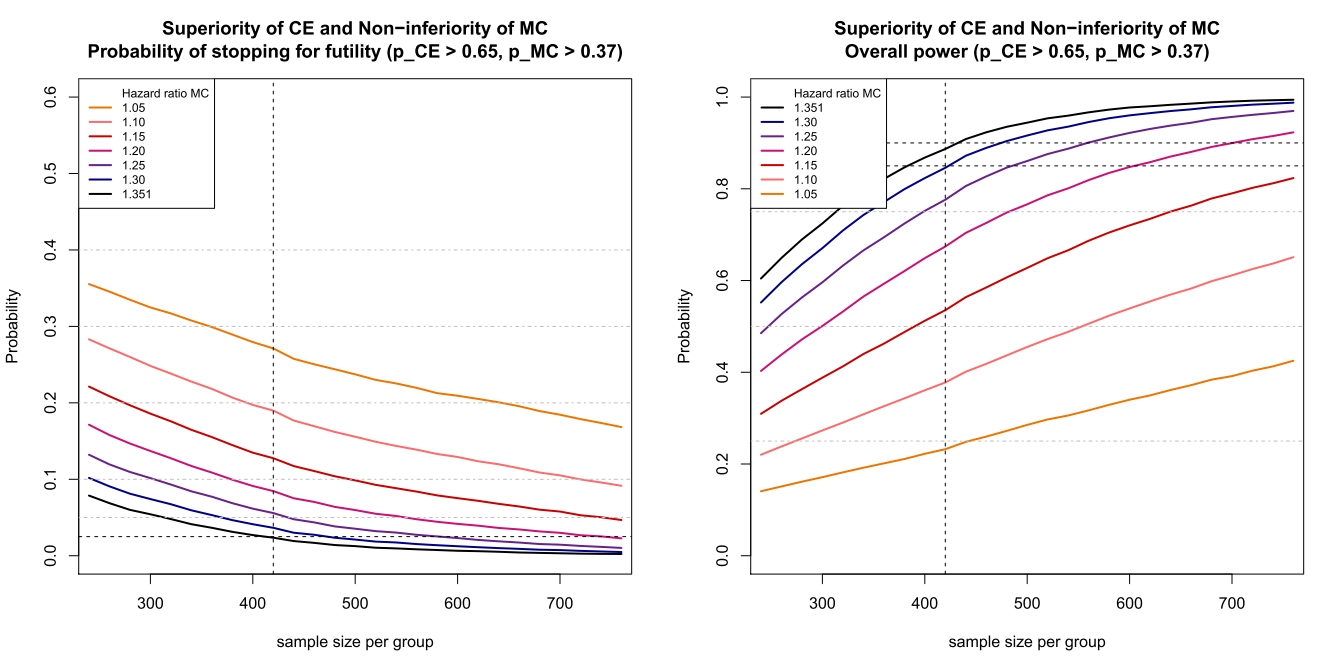

Supplement: Supplementary file 3 — Probability of stopping for futility and overall power for Approach 2. Plot of probability of stopping for futility (left figure) and overall power (right figure) for Approach 2 using the futility β γ-boundaries $\alpha_{f}^{CE}=0.65$αfCE=0.65 and $\alpha_{f}^{MC}=0.37$αfMC=0.37 for composite endpoint and main component, respectively. (JPG 238 kb) [file 12874_2017_387_MOESM3_ESM.jpg]

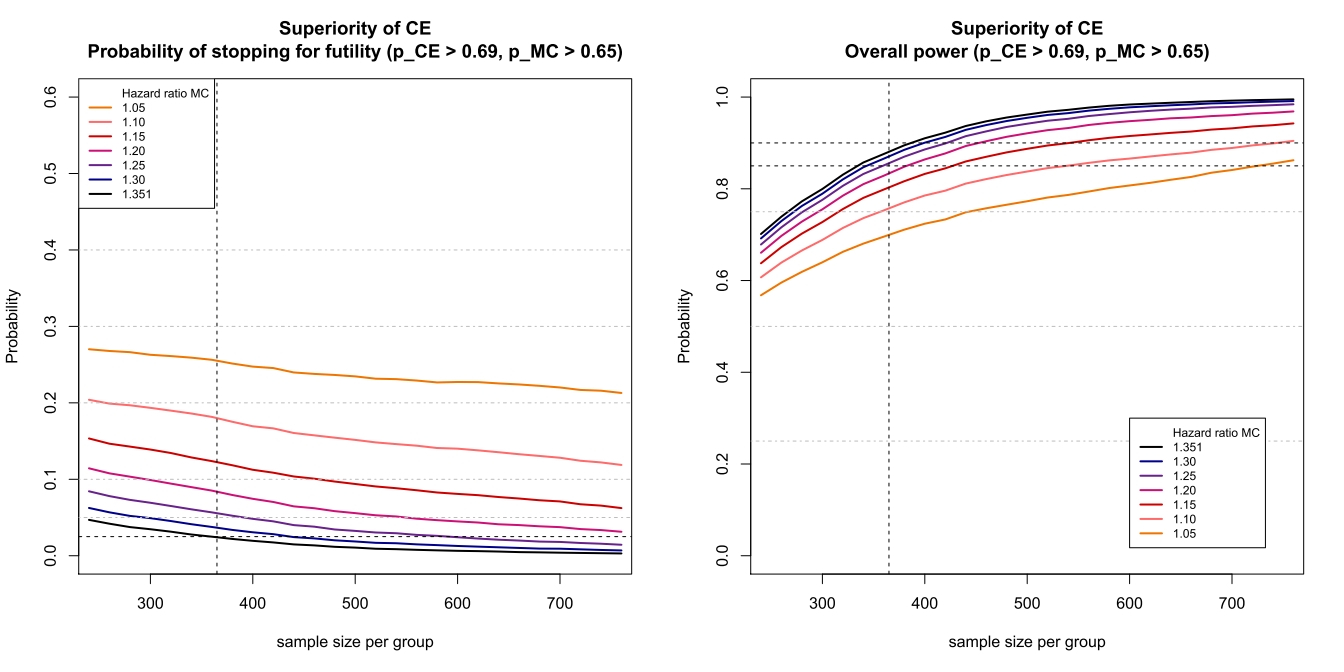

Supplement: Supplementary file 4 — Probability of stopping for futility and overall power for Approach 3. Plot of probability of stopping for futility (left figure) and overall power (right figure) for Approach 3 using the futility β γ-boundaries $\alpha_{f}^{CE}=0.69$αfCE=0.69 and $\alpha_{f}^{MC}=0.65$αfMC=0.65 for composite endpoint and main component, respectively. (JPG 202 kb) [file 12874_2017_387_MOESM4_ESM.jpg]
